# UGT1A1 sequence variants and bilirubin levels in early postnatal life: a quantitative approach

**DOI:** 10.1186/1471-2350-12-57

**Published:** 2011-04-22

**Authors:** Neil A Hanchard, Jennifer Skierka, Amy Weaver, Brad S Karon, Dietrich Matern, Walter Cook, Dennis J O'Kane

**Affiliations:** 1 Department of Laboratory Medicine and Pathology, Mayo Clinic and Foundation, 200 1stSt SW, Rochester, MN, 55905, USA; 2 Department of Pediatric and Adolescent Medicine, Mayo Clinic and Foundation, 200 1stSt SW, Rochester, MN, 55905, USA; 3 Department of Medical Genetics, Baylor College of Medicine, One Baylor Plaza, Houston, TX 77030, USA

## Abstract

**Background:**

Fundamental to definitively identifying neonates at risk of developing significant hyperbilirubinemia is a better understanding of the genetic factors associated with early bilirubin rise. Previous genetic studies have focused on the UGT1A1 gene, associating common variation in the coding or promoter regions with qualitative assessments of bilirubin (i.e. significantly elevated or not). These studies have had conflicting results and limited success. We chose to approach the problem by focusing on the quantitative (absolute) change in bilirubin levels early in post-natal life. We apply this approach to the UGT1A1 gene - exploring the contribution of both rare and common variants to early bilirubin changes.

**Methods:**

We sequenced the exons, PBREM, 5'-, and 3'- regions of the UGT1A1 gene in 80 otherwise healthy term neonates who had repeat bilirubin levels measured within the first five days of life.

**Results:**

Three novel coding variants were observed, but there was no clear relationship between rare coding variants and bilirubin rise. Adjusted linear regression models fit to evaluate the relationship between changing bilirubin levels and common UGT1A1variants found that among 39 neonates whose bilirubin was resampled within 33 hours, individuals homozygous for the mutant allele of a 3'UTR SNP had significantly smaller changes in bilirubin (P = 0.003) than individuals carrying the wild-type allele.

**Conclusions:**

Collectively, rare UGT1A1 coding variants do not appear to play a prominent role in determining early bilirubin levels; however common variants in the 3' UTR of UGT1A1 may modulate the early bilirubin rise. A quantitative approach to evaluating early bilirubin kinetics provides a more robust framework in which to better understand the genetics of neonatal hyperbilirubinemia.

## Background

Neonatal hyperbilirubinemia affects 60% of full-term newborns and remains a significant cause of hospital re-admission in the first week of life [1,2]. With most newborns being discharged from hospital at 48 hours of life - a time when serum unconjugated bilirubin levels are still rising - the American Academy of Pediatrics has placed more emphasis on identifying infants at increased risk of developing significant hyperbilirubinemia [3]. These infants require closer follow-up to stave off the potentially devastating neurological effects of bilirubin encephalopathy.

In practice, an otherwise healthy term newborn's risk for developing severe 'physiological' hyperbilirubinemia is largely a function of their bilirubin level at discharge. Bilirubin, when plotted on an hour-specific nomogram, often directs the need for future evaluation [4-6]. Among those identified as being at high or high-intermediate risk at discharge, however, only 8% will subsequently develop significant hyperbilirubinemia [6]. One study estimated the positive predictive value of the discharge bilirubin at 48% [7], underscoring the difficulty in predicting bilirubin rise during the first week of life. Ideally, a simple combination of clinical and laboratory information would definitively identify those infants whose bilirubin levels are likely to rise substantially, while minimizing the associated costs and anxiety to those with lower risks. Fundamental to this goal is a better understanding of the factors that determine the early rise of bilirubin.

The production and degradation of bilirubin is multifactorial and complex, involving both maternal and newborn factors as well as innate genetic factors (see [8]). Recent data, however, suggests that assessing additional maternal and neonatal factors beyond discharge bilirubin level and gestational age, does not significantly improve the accuracy of risk stratification [6]. This implies that innate or genetic factors may play an important role in determining the rise of bilirubin in the first week of life. The rate limiting enzyme in the hepatic conjugation of bilirubin is UDP-glucoronosyl transferase A1 (UGT1A1)[9], the activity of which is controlled by variants in the gene of the same name (OMIM - 191740).

Common variants in UGT1A1 have long been thought to influence the occurrence of severe physiological hyperbilirubinemia in neonates [10]; however, the multifactorial nature of the disorder has made it difficult to decipher this relationship. For example, a common missense mutation at position 211 in exon one of UGT1A1 has been consistently associated with significant and persistent hyperbilirubinemia in neonates of East Asian ethnicity [11-13], but is relatively rare in Caucasian or African populations [14]. In the latter populations, the common TA repeat motif in the 5-prime (5') promoter region, associated with the Gilbert syndrome phenotype in later life [15], has been intensely scrutinized; possession of 7 or more TA repeats in combination with G-6-PD deficiency has been associated with significant hyperbilirubinemia in neonates [16,17]. A similar number of repeats has also been reported in association with both an increase in bilirubin over the first 48 hours of life [18] and a slower return to baseline bilirubin levels [19].

Despite this, the precise role of UGT1A1 in exaggerated physiological hyperbilirubinemia remains equivocal; subsequent studies have been unable to replicate many of the above associations [20,21], while other potentially associated regions such as the upstream Phenobarbital response enhancer module (PBREM) [22], a regulator of UGT1A1 activity, has scarcely been evaluated [23]. Most recently, Watcho and colleagues [24] evaluated the role of eleven common, previously described UGT1A1 variants in a case-control study of severe hyperbilirubinemia (defined as having a peak bilirubin level > 95^th ^percentile). They were unable to find statistically significant differences in the frequency of any of the UGT1A1 variants between cases and controls; citing the ethnic diversity of the sample groups as a possible confounder, and conceding that a comparison of neonates with bilirubin levels above the 95th percentile versus those with levels below the 40th percentile may not be a robust discriminator of genotypes related to hyperbilirubinemia.

Given this complexity, we considered whether a study designed around the clinical conundrum of predicting the change in bilirubin levels, rather than the absolute occurrence of significant hyperbilirubinemia, might lead to a better understanding of early bilirubin kinetics. This approach can substantially improve the statistical power of genetic studies [25], and is being used in the current wave of genome-wide association studies [26]. Along with this, we also wished to interrogate new and as yet untested variants within UGT1A1. To explore the dynamics of this approach, we performed a pilot study of quantitative changes in bilirubin designed to identify potential susceptibility loci influencing early bilirubin levels. We sequenced the UGT1A1 gene, including the PBREM region, the 5' and 3' untranslated regions (UTR's), and all five exons, in a population of eighty otherwise healthy term neonates who had repeated bilirubin evaluations in the first 5 days of life.

## Methods

### Study Subjects

Study participants were chosen retrospectively from among a cohort of neonates born at the Rochester Methodist Hospital, Mayo Clinic in Olmsted County, Minnesota. These neonates were all enrolled in a previous study of transcutaneous bilirubin estimation carried out at our institution [27]. Briefly, this included 178 non-consecutive, near-term to term (gestational age greater than 35 weeks), otherwise healthy newborns who were thought to be clinically jaundiced prior to discharge from the newborn nursery. The parents of all participants consented to their newborn undergoing a measurement of a transcutaneous bilirubin alongside the usual serum bilirubin measurement. Each neonate's gestational age at birth, postnatal age at time of serum bilirubin measurement, and mother's ethnicity was recorded at the time of initial enrollment.

From this cohort, we obtained permission to review the medical record in order to determine those individuals who, as a matter of clinical course, required a serum bilirubin to be repeated within 120 hours of life. Thus all included participants would have had serum bilirubin measurements done prior to discharge and again within the first five days of life. These serum measurements were the only bilirubin measurements used in the study. Any participant who had a medically significant intervention (including phototherapy) done prior to their repeat serum bilirubin, as well as those with a positive Coombs test (where one was done), gestational age less than 36 completed weeks, or a birth weight less than 2.5 kg, were excluded from further consideration. For the remaining 80 participants, details of weight at discharge from hospital, medical illnesses (including a positive newborn screen), and timing of samples were extracted from the medical record. The final cohort did not differ significantly in demographic make-up from the original cohort. Clinical and laboratory variables at the time of each serum bilirubin measurement are given in Additional file 1: Supplemental Table s1.

The study design was approved by the Institutional Review Board of the Mayo Clinic.

### Gene sequencing

Newborn screening cards (dried blood spots - DBS) for each individual, initially obtained through informed consent, were randomly assigned a unique identifier linking the collated clinical information to the respective blood spot, but untraceable to clinical identifiers.

DNA was extracted from each DBS using the EZ1 BioRobot (QIAGEN^®^). Direct DNA sequencing was performed according to previously published methods [28]. The non-coding DNA sequences (introns) were not sequenced due to their very large sizes. Mutation Surveyor^® ^(SoftGenetics^®^, LLC, State College, PA), was used to identify polymorphic variants from the sequence data. Identified polymorphisms were then confirmed or rejected through concordance between two observers (NH and JS).

Recorded variants were then matched to the public SNP database where possible, using dbSNP build 129 (http://www.ncbi.nlm.nih.gov/projects/SNP/). One SNP (rs11568318) of minor allele frequency (MAF) 0.02 was found to be out of Hardy-Weinberg equilibrium (P < 0.01 by chi-squared) and was excluded from further study.

### Linkage disequilibrium mapping and haplotype construction

For the purposes of linkage disequilibrium mapping and haplotype construction, we chose to exclude variants with MAF < 0.10 since such SNPs are unlikely to be informative in a sample sizes such as ours. Pairwise estimates of linkage disequilibrium using absolute values of D' were calculated and graphically displayed using the MARKER software (http://www.gmap.net/marker). Haplotypes were statistically inferred using the algorithm found in PHASE version 2.1[29].

### Statistical analyses

Separate linear regression models were fit to evaluate the relationship between the change in bilirubin level (mg/dl) (second assessment minus first assessment) and demographic or genetic factors. Each model was adjusted for age (in hours) at the first bilirubin assessment, the level of the first bilirubin assessment, and the time interval (in hours) between the two assessments. The relationships were summarized by reporting the parameter estimates, with corresponding ninety-five percent confidence intervals (95% CI), and p-values from the adjusted linear regression models. All calculated p-values were two-sided and p-values less than 0.05 were considered statistically significant. Analyses were performed using the SAS version 9.0 software package (SAS Institute, Cary, NC).

## Results

### Demographic factors

We first sought to determine whether any of the demographic or clinical characteristics of the study cohort had an influence on the rise of bilirubin with time using our adjusted linear regression models (see methods). Under this model, gestational age (per week of maturity) showed a significant negative association with the change in bilirubin (Parameter estimate [slope] = -0.60 [95% CI, -0.99 to -0.21]; P = 0.003), i.e. for each additional week of gestational age at birth, the mean change in bilirubin between the two samples was 0.60 mg/dl lower. Percent weight loss demonstrated a trend toward a larger increase in bilirubin with greater degrees of weight loss, but this did not reach statistical significance (Parameter estimate (slope) = +0.17 (95% CI, -0.02 to 0.37); P = 0. 075). Factors common to the cohort, such as breastfeeding and maternal Caucasian ethnicity, were not significantly associated with change in bilirubin, either before or after adjustment. The details of the clinical factors evaluated are given in Table 1.

**Table 1:** Demographic variables.

Variable	Total (N = 80)
**Gender**	
Female	41 (51.3%)
Male	39 (48.8%)
**Ethnicity**	
Caucasian	68 (85%)
Asian	8 (10%)
Hispanic	3 (3.8%)
Black	1 (1.3%)
**Feeding method**	
Breast	71 (88.8%)
Bottle	5 (6.3%)
Both	4 (5%)
**Gestational age (wks)**	
Median	39.0
Interquartile range	37.7, 39.7
Range	36.4 - 41.4
**Gestational age**	
36 to < 38 weeks	23 (28.8%)
≥ 38 weeks	57 (71.2%)
**Coombs status**	
Negative	15 (18.7%)
Unknown	65 (81.3%)
**Birth weight (kilograms)**	
Mean (SD)	3.4 (0.4)
Range	2.5 - 4.5
**Percent weight loss at discharge**	
Mean (SD)	6.1 (2.6)
Range	-1.3* - 13.1

* Negative percent weight loss represents weight gain.

### Genetic factors

A total of 26 UGT1A1 gene variants were identified in the cohort (Table 2). All were single base substitutions except the previously described TA insertion site [15]. Twelve variants were represented in the UGT1A1 allele database [30].

**Table 2:** Sequence variants observed in UGT1A1.

**dbSNP no**.	* Allele/SNP alias	**Chr. pos**.	Major allele	Minor allele	MAF
rs11568318	*89	234330237	C	A	0.02
**rs4124874**	*60	**234330398**	**T**	**G**	**0.40**
rs11568319	*91	234330500	C	G	0.01
rs11568317	*92	234330502	A	G	0.01
-	-3162	234330515	G	C	0.01
**rs10929302**	*93	**234330521**	**G**	**A**	**0.29**
-	-136	234333537	C	T	0.01
rs873478	*81	234333609	G	C	0.01
**rs34815109**	*28/(TA) rpt	**234333620**	**6**	**7**	**0.32**
rs4148323	*6	234333883	G	A	0.01
-	+341	234334012	A	G	0.01
-	+449	234334120	C	T	0.01
-	+540	234334211	A	G	0.01
rs6708136	-	234340306	C	T	0.03
rs28946890	-	234341112	T	C	0.01
rs34082659	-	234341148	C	T	0.02
**rs2302538**	*66/*88	**234341152**	**T**	**C**	**0.10**
rs12471326	-	234341197	T	C	0.04
-	+12724	234341376	A	G	0.01
**rs10929303**	*76	**234346155**	**C**	**T**	**0.19**
**rs1042640**	*78	**234346283**	**C**	**G**	**0.17**
**rs8330**	*79	**234346384**	**C**	**G**	**0.21**
-	+17781	234346539	G	T	0.01
**rs17862880**	-	**234346795**	**C**	**A**	**0.10**
**rs4148329**	-	**234346801**	**C**	**T**	0.65*
-	+18162	234346815	A	G	0.01

Chromosome position (Chr. pos.), dbSNP numbers, and minor allele are referenced to dbSNP build 129. Where there was no previous description for an allele, the approximate SNP position referenced from the ATG start site was used. Approximate minor allele frequencies (MAF) are given in the final column with alleles of MAF ≥ 0.10 used for subsequent association study given in bold. * - dbSNP minor allele given as T; in our sample the C allele was more common.

#### Coding variants

Table 3a gives the molecular details of the identified coding variants. Five individuals (6.3%, 95% CI 2.1% -14.0%) were heterozygous at four loci in the first exon of UGT1A1. Three of these variants had not been previously described (positions +449, +341 and +540) and of these, two coded for non-synonymous (missense) amino acid changes. Three variants were found in association with either a heterozygous (6/7) or mutant homozygous (7/7) variation of the TA promoter repeat motif.

**Table 3:** Observed UGT1A1 coding variants.

3a										
**Individual**	**Chr. pos**.	**Exon**	**Gene pos**.	**AA pos**.	**SNP change**	**(TA) rpt**	**Type**^**a**^	**original AA**	**new AA**	

MS003	234334120	1A	449	150	C → T	6/7	N/S	Threonine	Methionine	
MS036	234333883	1A	211	71	G → A	6/6	N/S	Glycine	Arginine	
MS057	234333883	1A	211	71	G → A	6/7	N/S	Glycine	Arginine	
MS039	234334012	1A	341	114	A → G	7/7	N/S	Lysine	Arginine	
MS081	234334211	1A	540	180	A → G	6/6	S	Glutamic Acid	Glutamic Acid	

**3b**										

**Individual**	**Maternal ethnicity**	**GA**	**% wt loss**^**b**^	**Feeding method**	**Initial bilirubin**	**Initial risk zone**^**c**^	**Repeat bilirubin**	**Repeat risk zone**^**c**^	**Bilirubin increase (mg/dl per hr)**	**Required Phototherapy**

MS003	Caucasian	37.0	6.4%	Breast	10.2	LI	16.9	High	0.29	Yes
MS036	Asian	37.0	7.7%	Breast	13.6	High	20.9	High	0.16	Yes
MS057	Asian	37.4	-0.4%	Bottle	12.5	LI	15.0	HI	0.16	No
MS039	Caucasian	39.4	3.1%	Bottle	11.3	HI	10.3	Low	-0.02	No
MS081	Caucasian	39.9	4.9%	Breast	12.2	HI	14.9	HI	0.10	No

Table 3a gives the molecular characteristics of the observed variables for each individual. Chromosome position (Chr. pos.), Gene position (Gene pos.) and amino acid (AA) position (pos.) are given with reference to dbSNP build 129. ^**a**^N/S = non-synonymous substitution; S = synonymous substitution. Table 3b gives the associated clinical characteristics for each individual. ^**b**^negative percent weight loss (% wgt loss) is a gain of weight. ^**c**^nomogram based risk zones; LI = low intermediate; HI = high intermediate.

Clinically, four of the five individuals with coding variants had an initial bilirubin that placed them in the high or high-intermediate risk zone (Table 3b). Despite this, only one of these individuals (MS003) had a rate of rise that was outside the sample distribution for increasing bilirubin, i.e. more than two standard deviations above the mean per-hour increase (mean (SD), 0.09 (0.08) mg/dl/hr), and only two individuals eventually required phototherapy for significant hyperbilirubinemia; both individuals were born at 37 weeks gestation. We also examined the four other individuals in the broader cohort who had a rate of bilirubin rise (mg/dl/hour) greater than two standard deviations; one had a rare singleton allele of uncertain significance in the 3'UTR (+18162), otherwise there was no clear relationship between possession of a rare variant (coding or non-coding) and an increased rate of bilirubin rise, irrespective of TA repeat status.

#### Common non-coding variants

Using adjusted linear regression models (see methods), we evaluated the effect of common (MAF > 10%; N = 9) variants on the rise of bilirubin. In our initial modeling we did not find any significant association between either genotype or allele and the change in bilirubin.

Bilirubin levels change in a non-linear fashion over the first five days of life, particularly over the last three days. As we had only two sample points over the entire time period, we surmised that there might be more uncertainty in the trajectory of bilirubin levels between sampling intervals that were more distant than those that were closer together. For those sampling intervals that were closer together, any change in bilirubin would appear more linear, thus better approximating the statistical model we were using. We found that the distribution of sampling intervals in the cohort was bimodal (Additional file 2: Supplemental figure f1a) with median 33 hours, and that the change in bilirubin was more uniform (slope of zero) across the group sampled at shorter intervals, as opposed to the group sampled at greater intervals (Additional file 2: Supplemental figure f1b).

**Figure 1:**
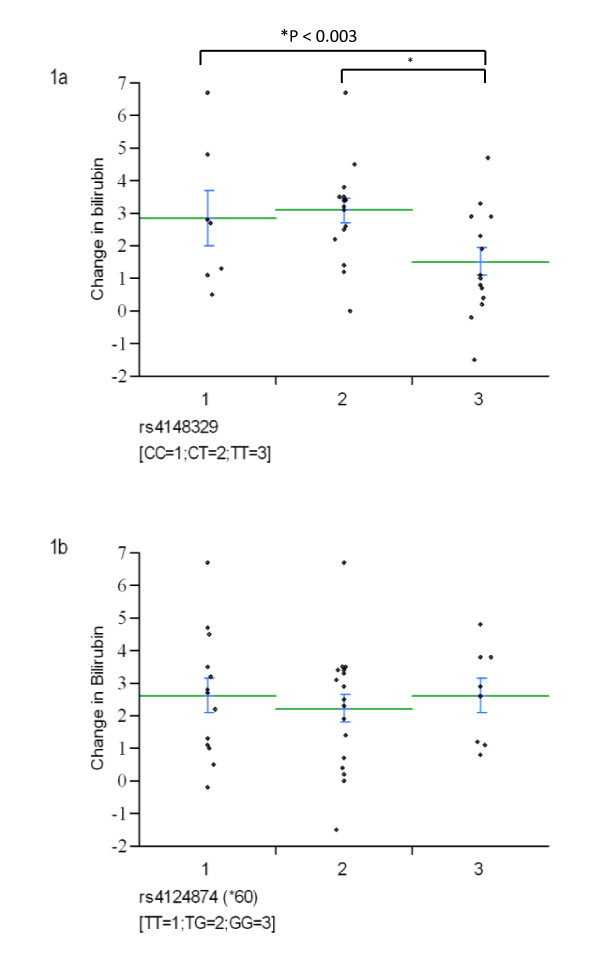
**Change in bilirubin level (between 1^st ^and 2^nd ^bilirubin measurements, mg/dl) by genotype for two UGT1A1 SNPs among neonates who had a repeat bilirubin measured within 33 hours**. Figure 1a illustrates the association with SNP rs4148329 - a smaller change is observed for neonates who were homozygous for the mutant allele versus those with wild-type genotypes. This was significant at the P < 0.003 level. A non-significant association with a SNP of comparable minor allele frequency is given in the lower figure (1b) for comparative purposes. In each figure the change in bilirubin for each neonate is given as a diamond; the green lines are the mean values for each genotype and the blue bars show the standard error of the mean.

We thus performed a sub-group analysis in which we focused on those individuals who had their bilirubin re-sampled within 33 hours (N = 39). The first step in this analysis was to graphically evaluate whether there was any visual difference in the mean change in bilirubin between subjects having different genotypes. This approach suggested a lower mean change in bilirubin of subjects having the mutant (TT) genotype at SNP rs4148329 versus persons with the wild-type homozygous or heterozygous genotype (mean change in bilirubin = 1.52 vs. 3.01 or 2.84 mg/dl, for TT vs CT or CC - Figure 1). When we applied our adjusted regression model to this subgroup analysis, this difference was significant under this recessive disease model (i.e. TT vs CT or CC) (difference in adjusted mean change in bilirubin = -1.78 (95% CI -2.90 to -0.66); P = 0.003), and remained significant after accounting for the effects of gestational age (P = 0.002). None of the other eight SNPs showed any evidence of significant differences in mean bilirubin level.

### Linkage disequilibrium and haplotype analysis

**Figure 2:**
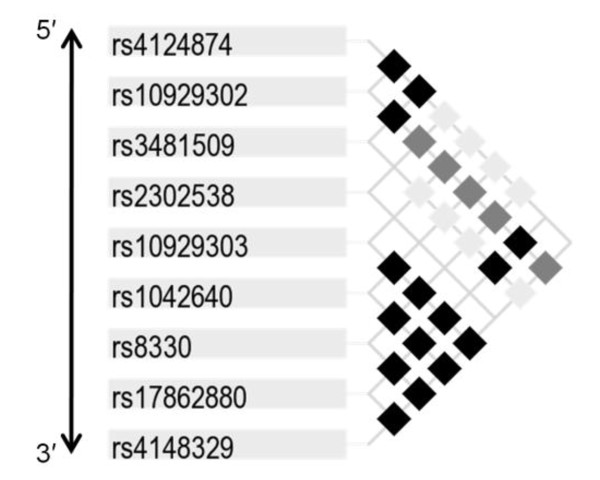
**Pair-wise linkage disequilibrium (LD) across UGT1A1 using common markers (minor allele frequency ≥ 10%)**. Black boxes indicate pair-wise D' values ≥ 0.9; dark gray boxes indicate D' values > 0.7 but < 0.9; light gray boxes indicate D' values between 0.5 and 0.7; D' values < 0.5 are indicated by a cross. Two 'blocks' of strong LD (D' ≥ 0.9) are seen at the 5' and 3' ends.

In order to further appraise the contribution of common variants to the rise of bilirubin, we also performed a similar analysis using haplotypes inferred across the UGT1A1 gene. We approached this by first determining pair-wise linkage disequilibrium across the region. This demonstrated two 'blocks' of strong linkage disequilibrium - defined as D' between markers of 0.9 or more- one in the 5' region inclusive of markers between the *60 allele and the TA repeat motif, and another in the 3' region extending from the *76 allele to rs4148329 (Figure 2). We then constructed haplotypes (see Methods) in each of the two regions and repeated our multivariate analysis. For this analysis, haplotypes with frequency less than 10% were not included. The mean change in bilirubin was then analyzed among carriers of each haplotype (without respect to hetero- or homo-zygosity) versus non-carriers of the haplotype under test.

In the adjusted linear regression analysis using a similar sub-grouping (bilirubin re-sampled less than 33 hours later), none of the commonly observed haplotypes were associated with a significant change in bilirubin (Additional file 3: Supplemental Table s2).

## Discussion

This is the first study of early bilirubin rise to sequence the PBREM and 3 untranslated region in additional to the exons and 5' promoter of UGT1A1 in a Caucasian sample. We found novel coding SNPs in exon one that occurred independently of significant early increases in bilirubin. We also found a SNP in the 3' untranslated region of the gene that, on subgroup analysis, showed an inverse relationship with bilirubin rise; this was independent of gestational age, which was also inversely associated with greater rises in bilirubin. Most importantly, this study provides a unique framework in which to further evaluate the role of UGT1A1 variants in early bilirubin rise.

Any evaluation of neonatal hyperbilirubinemia, genetic or otherwise, will necessarily include clinical parameters usually evaluated in jaundiced neonates. In keeping with the fairly homogenous ethnic distribution of Olmsted County, most mothers reported their ethnicity to be Caucasian. This reduces the impact of population stratification inherent in the incidence and etiology of neonatal hyperbilirubinemia, and accordingly, our major conclusions did not change when we excluded those neonates with non-Caucasian ethnicity (data not shown). Most neonates in our cohort were also breastfed, reducing the likelihood that observed differences in hyperbilirubinemia were the result of differences in feeding preferences. We found gestational age (per week of increased maturity) to be inversely associated with an increase in bilirubin. This is consistent with larger studies of clinical factors affecting neonatal hyperbilirubinemia [6]. We also found a trend toward a positive association between weight loss at discharge (per 1% loss) and a greater rise in bilirubin, which likely reflects the known effect of breastfeeding and decreased volume intake in the initial days after birth.

Sequencing the UGT1A1 gene allowed us to evaluate the collective effect of rare coding variants on early bilirubin rise. The rare variation observed was limited to exon one, consistent with the conserved nature of exons 2 to 5, which are common to all UGT1 genes; three of the four coding SNPs observed have not, to the best of our knowledge, been previously described. The variant at position +449 encodes a non-synonymous amino acid change from Threonine to Methionine. This is an unusual substitution that results in a change from a hydrophilic to a hydrophobic amino acid. Although the individual with this change was in the low-intermediate risk zone at the time of discharge, the subsequent rate of rise of bilirubin over time was more than two standard deviations beyond the distribution of the cohort, and resulted in subsequent re-admission for phototherapy after repeat sampling. Despite this, there was no definite relationship between possession of a heterozygous coding SNP and hyperbilirubinemia. For instance, the well-described *6 allele commonly found in persons of East-Asian ethnicity, was found in conjunction with the supposedly protective wild-type TA repeat (6/6), and this individual was not only at high risk at the time of discharge, but had a sharp increase in bilirubin and ultimately required phototherapy; conversely another individual with the *6 allele was heterozygous at the TA repeat (6/7), was low-intermediate risk at birth, and did not require any intervention. This illustrates the imperfect relationship between coding variants and early bilirubin levels and underscores the complex multifactorial nature of the disease.

When we focused on the common variants observed in the cohort, we found that among neonates whose bilirubin was re-sampled at less than 33 hours, homozygotes for the mutant 'T' allele of the rs4148329 SNP had a significantly smaller (mean) change in bilirubin than either homo- or heterozygotes of the wild-type allele. Our evaluation of the 3'-UTR is novel among studies of neonatal hyperbilirubinemia, thus a putative role for rs4148329 is unclear; it encodes a predicted mRNA regulatory binding site in the 3' -UTR for which there is no current functional data and there are no previous associations establishing it's role in hyperbilirubinemia. Recent work [31], however, suggests that the 3'-UTR is more complex than hitherto thought, including the description of alternate splice variants of the last exon of UGT1A1 that encode widely-distributed isoforms of UGT1A1 that are functionally divergent. The 3'-UTR of UGT1A1 thus represents an interesting and emerging area that warrants further investigation. In the absence of such studies, however, any definitive conclusion about the role of rs4148329 in the early rise of bilirubin remains speculative.

The retrospective sampling that shaped the clinical distribution of the cohort and the sample size employed in the study meant that our pilot protocol was primarily geared toward looking at the collective role of rare, rather than common, UGT1A1 variants. We did not find strong evidence for the role of common 5'-promoter variants, including the Gilbert syndrome (TA) repeat motif, in early bilirubin rise in this population. This may be reflective of the study population, the quantitative approach taken, or the modest sample size employed, which limited our ability to definitively identify common variants with relatively small effect sizes of the kind expected for the Gilbert genotype. The linkage disequilibrium pattern observed also underscores the complexity of studying genetic susceptibility to neonatal hyperbilirubinemia; although we limited our appraisal to common population variants, we still observed strong LD between markers in the 5'  and 3'  regions respectively. This strong LD suggests that refining association signals found in either region will be challenging, and may explain the difficulty in replicating previous associations across differing populations with differing local LD patterns.

Our quantitative approach to hyperbilirubinemia is somewhat unique, and despite the exploratory nature of this pilot study, it provides a framework for a larger and more comprehensive evaluation of susceptibility genes in the early rise of bilirubin. The quantitative trait approach allowed us to evaluate a broad range of changes in bilirubin as opposed to the qualitative outcome of 'significant hyperbilirubinemia' that has been the focus of previous studies. For instance, our SNP association suggests that there may be variants that *attenuate *the early rise in bilirubin; this may not be readily apparent with a qualitative approach in which such observations are unlikely to be represented. Our study also suggests that, ideally, individuals should be sampled at regular intervals over the first few days of life. This would circumvent the non-linearity of bilirubin rise and facilitate the use of a more robust statistical model to better exploit the inherent power advantages of a quantitative approach. Finally, it is worth noting that a quantitative approach could be undertaken in conjunction with a genome-wide SNP scan; such genome-wide association studies have been utilized to identify genetic determinants of bilirubin levels in adults [32,33], and could ultimately lead to a better understanding of the genetic factors controlling the early neonatal bilirubin rise.

## Conclusions

Our results suggest that rare coding variants do not collectively play a significant role in the early rise of bilirubin, while common variants in the hitherto unevaluated 3'UTR may modulate early bilirubin rise in this population. A quantitative approach to the evaluation of early bilirubin changes may provide a more robust statistical framework in which to determine genetic variants that both augment and attenuate physiological *ex-utero *changes in bilirubin.

## Competing interests

The authors declare that they have no competing interests.

## Authors' contributions

JS was involved in the conception and implementation of the study, including its technical execution. AW provided statistical analyses. WC and BK were involved in sample collection, and contributed to the writing of the manuscript. DM identified suitable DNA samples, and contributed to the study design and manuscript writing. DK was involved in the conception, implementation and administration of the study. NH conceived and designed the study, performed technical aspects and data analysis, and drafted the manuscript. All authors read and approved the final manuscript.

## Pre-publication history

The pre-publication history for this paper can be accessed here:

http://www.biomedcentral.com/1471-2350/12/57/prepub

## Supplementary Material

Additional file 1 **Sampling time characteristics**. Summary of clinical and bilirubin characteristics at each sampling time. Click here for file

Additional file 2 **Bilirubin by sampling interval**. Bilirubin levels and change in bilirubin for individuals sampled less than and more than 33 hours post first sample. Click here for file

Additional file 3 **Common-haplotype associations by region**. Haplotype frequencies, parameter estimates, and P-values for common haplotypes inferred in the proximal (5') and distal (3') portions of UGT1A1. Click here for file
