# Supplementary material for: UGT1A1 sequence variants and bilirubin levels in early postnatal life: a quantitative approach
Source: BMC Med Genet. 2011 Apr 22;12:57. doi: 10.1186/1471-2350-12-57 (PMC3107779; doi:10.1186/1471-2350-12-57)
Supplement: Additional file 1 — Sampling time characteristics. Summary of clinical and bilirubin characteristics at each sampling time. [file 1471-2350-12-57-S1.DOC]

**Supplemental Table s1**

| **Variable** | **1^st^ Bilirubin sampling** | **2^nd^ Bilirubin sampling** |
| --- | --- | --- |
| **Age (hours)** |  |  |
| Mean (SD) | 47.5 (11.7) | 82.9 (17.8) |
| Range | 24.0-80.5 | 40.0-118.5 |
|  |  |  |
| **Bilirubin (mg/dl)** |  |  |
| Mean (SD) | 10.5 (1.9) | 13.2 (2.6) |
| Range | 6.8-14.9 | 5.6-20.9 |
|  |  |  |
| **Risk zone** |  |  |
| Low | 4 (5%) | 17 (21.2%) |
| Low-intermediate | 38 (47.5%) | 30 (37.5%) |
| High-intermediate | 33 (41.2%) | 28 (35%) |
| High | 5 (6.3%) | 5 (6.3%) |

Summary of clinical and bilirubin characteristics at each sampling time (N = 80).
