# Supplementary material for: UGT1A1 sequence variants and bilirubin levels in early postnatal life: a quantitative approach
Source: BMC Med Genet. 2011 Apr 22;12:57. doi: 10.1186/1471-2350-12-57 (PMC3107779; doi:10.1186/1471-2350-12-57)
Supplement: Additional file 2 — Bilirubin by sampling interval. Bilirubin levels and change in bilirubin for individuals sampled less than and more than 33 hours post first sample. [file 1471-2350-12-57-S2.DOC]

**Supplemental Figure f1**

1a

1b

Bilirubin by sampling interval - Figure 1a shows bilirubin levels as a function of the time between sampling. Figure 1b shows the change in bilirubin (between the 1^st^ and 2^nd^ bilirubin measurements, mg/dl) in two sampling intervals – less than and more than 33 hours after 1^st^ sampling (1b). A line of best fit for each figure is given in black.
