# Supplementary material for: UGT1A1 sequence variants and bilirubin levels in early postnatal life: a quantitative approach
Source: BMC Med Genet. 2011 Apr 22;12:57. doi: 10.1186/1471-2350-12-57 (PMC3107779; doi:10.1186/1471-2350-12-57)
Supplement: Additional file 3 — Common-haplotype associations by region. Haplotype frequencies, parameter estimates, and P-values for common haplotypes inferred in the proximal (5') and distal (3') portions of UGT1A1. [file 1471-2350-12-57-S3.DOC]

**Supplementary Table s2**

|  | **HT label** | **HT** | **Freq.** | **Parameter** | **P-value** |
| --- | --- | --- | --- | --- | --- |
| **5′ Haplotypes** | 1 | TG(6) | 0.60 | -0.42 | 0.59 |
|  | 2 | GA(7) | 0.29 | +0.78 | 0.28 |
| **3′ Haplotypes** | 1 | CCCCT | 0.65 | -0.12 | 0.88 |
|  | 2 | CCCAC | 0.10 | -0.03 | 0.97 |
|  | 3 | TGGCC | 0.17 | +0.41 | 0.53 |

Common-haplotype associations by region - Haplotypes (HTs) are listed from 5′ to 3′ within each region (5′ haplotype - rs4124874 - rs10929302 - rs34815109/[TA rpt]; 3′ haplotype - rs10929303 - rs1042640 - rs8330 - rs17862880 - rs4148329).
